# Adolescent Obesity Modeling: A Framework of Socio-Economic Analysis on Public Health

**DOI:** 10.3390/healthcare9080925

**Published:** 2021-07-22

**Authors:** Hashem Salarzadeh Jenatabadi, Nurulaini Abu Shamsi, Boon-Kwee Ng, Nor Aishah Abdullah, Khairul Anam Che Mentri

**Affiliations:** Department of Science and Technology Studies, Faculty of Science, University of Malaya, Kuala Lumpur 50603, Malaysia; ainishamsi@um.edu.my (N.A.S.); bkng@um.edu.my (B.-K.N.); aishah.abdullah@um.edu.my (N.A.A.); khairulanam@um.edu.my (K.A.C.M.)

**Keywords:** household socioeconomic, adolescent obesity, public health, adolescent lifestyle, Bayesian structural equation modelling

## Abstract

Bayesian Structural Equation Modeling (SEM-Bayesian) was applied across different research areas to model the correlation between manifest and latent variables. The primary purpose of this study is to introduce a new framework of complexity to adolescent obesity modeling based on adolescent lifestyle through the application of SEM-Bayesian. The introduced model was designed based on the relationships among several factors: household socioeconomic status, healthy food intake, unhealthy food intake, lifestyle, body mass index (BMI) and body fat. One of the main contributions of this study is from considering both BMI and body fat as dependent variables. To demonstrate the reliability of the model, especially in terms of its fitting and accuracy, real-time data were extracted and analyzed across 881 adolescents from secondary schools in Tehran, Iran. The output of this study may be helpful for researchers who are interested in adolescent obesity modeling based on the lifestyle and household socioeconomic status of adolescents.

## 1. Introduction

The prevalence of obesity has almost tripled over the past four decades, and it is now identified as a global epidemic since it causes over four million deaths every year [1]. Indeed, this medical condition is a major threat to individuals’ health, as it increases the risk of developing various chronic diseases such as type 2 diabetes, non-alcoholic fatty liver disease, hypertension, stroke, obstructive sleep apnea (OSA) and several types of cancer [2]. Obesity can also result in a lower quality of life and unemployment, lower productivity and social disadvantages [3]. The World Health Organization (WHO) defines obesity as abnormal or excessive fat accumulation, which may impair health and is diagnosed at a BMI of ≥30 kg/m^2^. This health issue is prevalent across all ages, and WHO estimated that over 340 million children and adolescents were overweight or obese in 2016 [4]. Large bodies of research have also demonstrated that obese children and adolescents are much more likely to have elevated BMIs than adults [5].

Obesity does not only have adverse effects on adolescents’ physical health, but also their psychological health, as well as their academic achievement and social life. Research has indicated that, at schools, obese adolescents reportedly showed poor academic performance [6], had lower attendance rates [7] and were often involved in disciplinary offences [8]. Cate and Samouda [9] also found that behavioral and psychological problems among obese adolescents are higher compared to their non-obese counterparts. In terms of health-related quality of life (HRQoL), data consistently indicated that HRQoL decreases with an increasing level of BMI, where adolescents with extreme obesity were profoundly affected [10]. Furthermore, emerging evidence suggests that overweight or obese children and adolescents are particularly susceptible to psychosocial problems concerning self-image, body dissatisfaction, social stigmatization, peer victimization and depression [11]. In a recent study, Hales and Fryar [12] found that obesity among adults has risen dramatically since the 1980s, while it reached a plateau among adolescents between 2005–2006 and 2013–2014.

Quantitative analysis through systems modeling is particularly significant for advancing obesity studies, as it provides us with a better understanding of the correlations among various determinants of obesity. For example, statistical modeling serves as a tool in developing conceptual models that quantify the complex systems problem of obesity involving a wide range of social, economic, cultural, biological, environmental, behavioral and policy factors [13]. The model is widely used not only to inform policy planning and strategy at different levels of the government to overcome the complex issues of obesity among targeted population subgroups [14], but also serves as a basis for future research on the larger community to guide obesity prevention measures [15]. Economically speaking, this model has the potential to reduce the annual healthcare cost to society by suggesting efficient ways to prevent a further increase in obesity rates and improve its management [16]. Such preventive measures are particularly useful for researchers and decision-makers in public health systems. For example, one of the policy responses to the prevalence of obesity is the introduction of children and adolescents’ obesity intervention programs [17].

### 1.1. Previous Studies on Adolescent Obesity Modeling

Some research papers have justified and determined the indicators of obesity among adolescents, and in this study, we classified them into four main categories of main variables related to household environments: household socioeconomic; lifestyle; food intake (healthy food intake, unhealthy food intake); and parental obesity indices (parents’ BMI and parents’ body fat).

Previous studies on BMI indicated that household socioeconomic is the main key of household environment that has a significant impact on adolescent obesity, and this postulation has been proven by Mireku and Rodriguez [18] and Sigmund and Sigmundová [19] in their works. Meanwhile, another group of common indicators such as age, income, and education of the parents have also been examined in the previous empirical works on BMI. This paper integrates six variables (age of father, age of mother, education of father, education of mother, income of mother and income of father) with a latent construct (household socioeconomic) as the main independent variables.

Tee and Gan [20] confirmed that an unhealthy lifestyle is one of the main reasons for an adolescent being overweight or obese. Concerning the effect of an unhealthy lifestyle, some scholars found a relationship between physical activity and adolescents’ sleep behavior. To highlight this relationship, Micklesfield and Hanson [21] and Gillis and Shimizu [22] presented that poor sleep quality causes less physical activity among adolescents. Moreover, screen time reportedly has more impact on this relationship [23]. This claim was proven by Laurson and Lee [24], who indicated that a lack of exercise and sleep with increased screen time are the main reasons why adolescents become overweight and obese. Pocket money is another research variable that could have a direct or indirect impact on obesity. Bugge [25] in Norway found that different age groups spend their pocket money on unhealthy food, which indirectly affects the BMI level. Furthermore, smoking habits and drinking alcohol are indications of unhealthy lifestyles among adolescents [26]. However, this work informs us that more than 70% of the respondents were reluctant to reveal their smoking habits and alcohol consumption during the survey. Taking this into consideration, these two indicators are therefore omitted in our research framework. As a result, lifestyle in this study only takes four measurements into account: amount of sleep; pocket money; physical activity; and screen time.

Food intake behavior or food consumption is another main variable used in not only adolescent but also children, adult and elderly obesity modeling. Very few studies have involved one latent variable as food intake in their obesity modeling [27]; however, most of them applied both healthy food intake and unhealthy food intake separately in their obesity modeling [28,29]. In adolescent obesity modeling, sugar-sweetened, fast food and snacks are mostly included as the main unhealthy food consumption. For instance, Maruapula and Jackson [30] presented that many adolescents like to have snacks in their daily life. Another study by Sylvetsky and Visek [31] found that adolescents between 12–19 years old have the highest sugar-sweetened beverage consumption compared to other young age groups. In our study, we applied two latent variables of healthy food intake and unhealthy food intake, which are included among the seven measurement variables.

Finally, parents’ BMI is another measurement variable considered in some obesity modeling with [32,33] and without the SEM technique [34,35]. In this study, we involved parent’s BMI and parents’ body fat as the two control variables in our research framework. 

### 1.2. Research Framework and Contributions of the Study

**Figure 1:**
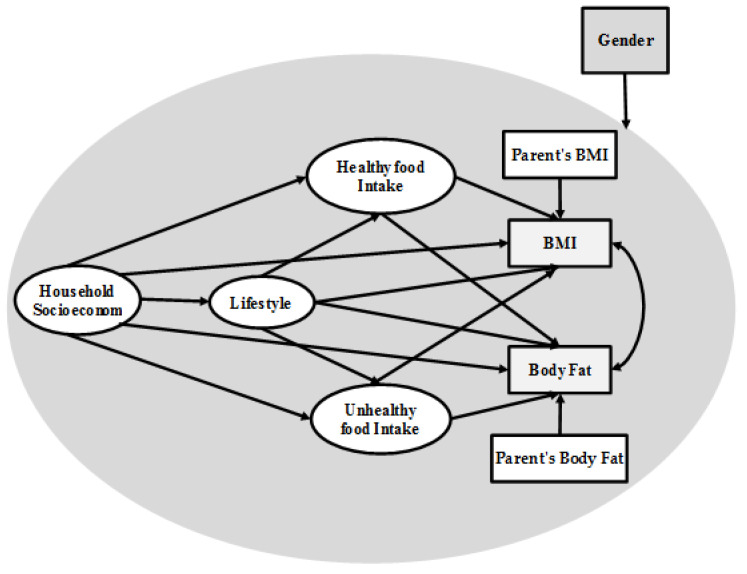
Research framework.

The proposed framework for investigating the respective relationships between these variables and adolescent obesity modeling is presented in Figure 1. In this framework, household socioeconomic status was selected as the independent variable, while the dependent variables include BMI and body fat. Three mediators were defined in the framework, i.e., healthy food intake, unhealthy food intake and lifestyle. Gender was also treated as a potential moderator in the associations between the variables and adolescent obesity modeling.

From a statistical and mathematical modeling perspective, correlation analysis, ANOVA, cross tabulation and regression are the most common methods for analyzing the associations between multiple variables and obesity. In recent decades, research scholars have presented specific concerns in obesity analysis using SEM. This statistical modeling technique allows for the estimation of BMI based on causal relationships among different types of measurement (observed) and latent (unobserved) variables. Maximum likelihood (ML) and partial least square (PLS) are the most commonly applied estimators in SEM analysis. Recently, however, some researchers have also applied the Bayesian estimator in the SEM technique. Nonetheless, some of the researchers are also conservative to discuss the distributions of variables. Therefore, without considering the distributions of research variables, they attempted using the non-parametric (free distribution) modeling. 

Muthén and Asparouhov [36] outlined the four key points that motivate the application of Bayesian estimator:

First point: It can analyze a new type of model-based approach. 

Second point: It can obtain good achievement with small-sample; researchers do not need any large-sample theory.

Third point: More can be learned about model fit and parameter estimates.

Fourth point: Analysis can be ended with fewer computational demands.

Bayesian analysis with SEM joint prior distributions for research parameters include the data likelihood to prepare prior distributions for estimating considered parameters. The prior might come from previous studies, and the posterior delivers an evaluation with the mean, mode or median form of posterior distributions. As per the parameters of interest, the Bayesian estimator is of concern, since it enables us to update current information in terms of prior information. To analyze the simulated data, we involved noninformative priors in this study. “Noninformative” specifies the prior distributions for the parameters, provided that little to no information and beyond the information was provided by the data [37]. In this situation, the posterior distributions are estimated around the information from the data instead of prior knowledge [38].

Since obesity is a multifactorial medical problem, this study focuses on several key factors, namely lifestyle, eating behavior and household socioeconomic status. The present study makes four important contributions to the existing literature on adolescent obesity modeling. First, the new model extends our understanding of the correlation between the critical determinants of BMI and body fat among adolescents. Second, this paper prepares a comparison analysis among three estimators of ML, PLS and Bayesian. Third, the present study provides a comparative analysis between the adolescent boy and girl obesity models using SEM-Bayesian with moderation analysis. Finally, the new model examines BMI and body fat as two dependent variables in a single analytical framework, which can provide a more comprehensive analysis of the real issues. However, this novel approach, which is crucial in providing insights into the complex network of factors, is lacking in the current literature on adolescent obesity modeling.

## 2. Materials and Methods

### Sampling Procedure

Several studies have highlighted the sample size issue in SEM. Gerbing and Anderson [39] believed that rational outcome could be contained in the SEM technique when the sample size is less than 200, while Boomsma [40] noted that the sample size should at least be more than 100. Furthermore, some statistics researchers have suggested applying the N:q ratio as a criterion for sample size with SEM analysis. The N:q ratio represents the number of participants (observation; cases) for each estimated parameter. However, there is a different suggestion for the N:q ratio by which Bentler and Chou [41] suggested 5 to 1, Schreiber and Nora [42] suggested 10 to 1, and Kline [43] suggested 20 to 1. In this study, we followed the N:q ratio based on Kline [43] since we have 17 parameters (household socioeconomics 6; lifestyle 4; healthy food 3; unhealthy food 4; parents’ BMI 1; parents’ body fat; BMI 1; Body fat 1; gender 1); thus, based on the 20:22 ratio, we required at least 440 participants. 

Hair and Black [44] also proposed significant terms and conditions for sampling in SEM. These are shown in Supplementary Table S1. In the present study, SEM was the lead for two subsections of the available data (adolescent boys and girls), and there were fewer than five latent variables of interest identified in both groups. The present study therefore required at least 200 respondents, and we subsequently designed a cross-sectional analysis for this study. From the statistical point of view, sampling with a cross-sectional design applies to any presumed research population samples at one point in time rather than over a period of time. Data for the present study were collected under the supervision of the Statistical Center of Iran. Fifteen undergraduate and master’s students of statistics, public health and management who did part-time jobs at the Statistics Department were trained for data collection. Participants were recruited from twenty-five private secondary schools in Tehran. Nested Sample or Multi-Stage Sample was also used for the sampling process. The Statistical Center of Iran later contacted the schools by email and telephone to explain the objectives of the project and seek collaboration on this study. Finally, out of 32 schools (with more than 300 students), 20 schools had agreed to collaborate on this project. The data were collected from the 20 schools between 10 January 2020 and 20–25 May 2020. We distributed fifty questionnaires (1000 in total) for each school and received 881 completed questionnaires, representing a response rate of 88.1%.

## 3. Results

### 3.1. Descriptive Statistics Analysis

Table 1 and Table 2 present the descriptive statistics of some major research variables. 

Based on Table 3, the largest percentage of mothers belongs to the age group between 31 and 40 years old (32.6%) and fathers in the group aged between 41 and 50 years old (36.2%). The largest percentage of individual income for both mothers and fathers was between 4 and 6 million Toman (28.9% for mothers and 36.2% for fathers). Both mothers and fathers mostly had a diploma (44%; 54.9%), while the largest percentage of job experience for both mothers and fathers was 11–15 years (39%; 45.8%).

As presented in Table 4, among adolescent boys, 28.1% of them had no physical activity at all, 38.3% had physical activity 1–2 times per week, 22% had physical activity 3–4 times per week, and 11.6% of them had physical activity more than 4 times per week. As for the physical activity among adolescent girls, 26.4% of them had no physical activity at all, 33.8% had physical activity 1–2 times per week, 29.3% had physical activity 3–4 times per week, and 10.4% of them had physical activity more than 4 times per week. Table 4 also shows no significant differences in terms of physical activity between adolescent boys and girls. However, the distributions of screen time between adolescent boys and girls were significantly different. Among adolescent boys, 1.9% of them had less than one hour of screen time per day, 26.7% of them had between 1 to 2 h per day, 27.4% had between 3 to 4 h per day and 44.1% of them had more than 4 h of screen time per day. Meanwhile, among adolescent girls, 2% of them had less than an hour of screen time per day, 11.3% had between 1 to 2 h per day, 44% had between 3 to 4 h per day and 42.7% of them had more than 4 h of screen time per day.

### 3.2. SEM Analysis

#### 3.2.1. Reliability and Validity Indices

Fornell and Larcker [45] proposed the terms and conditions for examining reliability and validity that include Cronbach’s alpha [should be higher than 0.7], average variance extracted (AVE) [should be higher than 0.5] and factor loadings [should be higher than 0.7]. Table 3 shows the outputs for factor loadings, AVE and Cronbach’s alpha. Factor loadings of “age of father”, “age of mother” and “income of mother” were less than 0.7; thus, these variables were eliminated from the rest of SEM-Bayesian analysis. However, all four latent variables had acceptable AVE and Cronbach’s alpha values; therefore, the reliability and validity of the research variables were confirmed.

#### 3.2.2. Fitting Model Analysis

**Figure 2:**
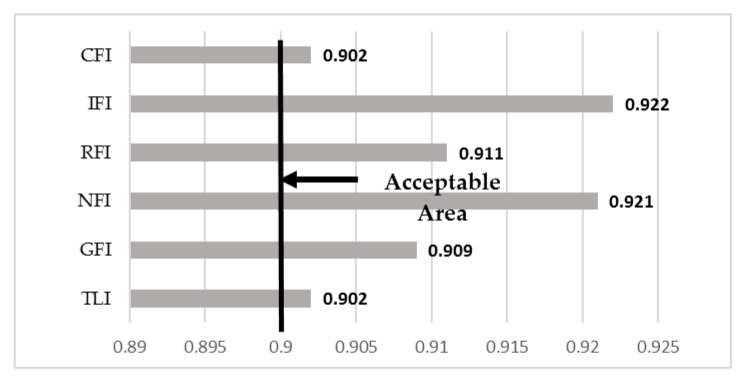
SEM model fitting.

Some statistical indices are frequently used in assessing model fit within SEM and these include GFI [goodness-of-fit index], NFI [normed fit index], IFI [incremental fit index], RFI [relative fit index], TLI [Tucker Lewis index] and CFI [comparative fit index]. Figure 2 shows the results for model fitting indices based on the SEM approach. The values of all indices were within acceptable range; therefore, the present framework as presented in Figure 1 was deemed a good fit for our data.

#### 3.2.3. Structural Model

Before presenting the structural model, we should first confirm which among the three estimators (PLS, ML and Bayesian) are more suitable for our data. Hence, four statistical indices were applied to compare the three estimators (See Table 4).

Based on Table 4, $y_{i}$ is the ith real value BMI and body fat (dependent variables) and $y_{i}^{,}$ is the *i*th predicted value of BMI and body fat. The R^2^ value for the SEM-Bayesian model was greater than the SEM-ML and SEM-PLS models, while the RMSE, MSE and MAPE values for the SEM-Bayesian model were lower than those of SEM-ML and SEM-PLS models. Therefore, based on the performance indices, SEM-Bayesian predicted BMI and body fat better than the SEM-ML and SEM-PLS models. However, this conclusion is only made for this empirical analysis, and does not prove that SEM-Bayesian is always superior to SEM-ML and SEM-PLS.

SEM-Bayesian is used by research scholars to drive posterior distributions, and perhaps some studies have also suggested applying the ML estimator to drive posterior distributions. Nevertheless, Lee and Shi [46] and Lee and Song [47] noted that high-dimensional integration is required in the SEM technique; therefore, it is not easy to involve the ML estimator, and as a result, they introduced the Gibbs sampler algorithm to overcome this issue. Briefly, the Gibbs sampler is a Markov Chain Monte Carlo (MCMC) method, which is based on creating a sequence of random observations from the fully conditional posterior distributions of unknown model parameters. 

Research involving the Bayesian estimator normally attempts to conclude priors such that they are informative enough to yield SEM-Bayesian advantages [48]. In this matter, Barroso and Roncancio [49] recommended sensitivity analysis where there is no security related to prior distributions. Therefore, in this stage of the study, we compared different prior outputs to test the influence of the priors. To accomplish this, we compared four models with different types of prior inputs. Lee [50] suggested assigning values to the hyperparameters by considering a small variance to each parameter. Therefore, we considered three prior inputs, which are computed accordingly as follows:

Type I Prior: considered to be 0.5 to all unknown loading coefficients. The estimate values related to $\left\{\beta_{1},\beta_{2},\beta_{3},\beta_{4},\beta_{5},\beta_{6},\;\beta_{7},\beta_{8}\right\}$ are {0.4,0.7, 0.3, 0.5, 0.7, 0.7, 0.6, 0,7} for the adolescent girl model and {0.5,0.4, 0.6, 0.6, 0.7, 0.7, 0.5, 0.6} for the adolescent boy model.

Type II Prior: The hyperparameter values are measured as half of the values in Prior I.

Type III Prior: The hyperparameter values are measured as double the values in Prior I.

Note: $\beta_{1}$ is the hyperparameter of Household Socioeconomic → BMI; $\beta_{2}$ is the hyperparameter of Household Socioeconomic → Body Fat; $\beta_{3}$ is the hyperparameter of Lifestyle → BMI; $\beta_{4}$ is the hyperparameter of Lifestyle → Body Fat; $\beta_{5}$ is the hyperparameter of Healthy Food Intake → BMI; $\beta_{6}$ is the hyperparameter of Healthy Food Intake → Body Fat; $\beta_{7}$ is the hyperparameter of Unhealthy Food Intake → BMI; $\beta_{8}$ is the hyperparameter of Unhealthy Food Intake → Body Fat.

Based on Table 5, the standard errors of estimated parameters under different types of prior distributions are close. This convinces us that statistical analysis with the SEM-Bayesian technique is not sensitive to the three hypothesized prior inputs. In other words, the modeling outputs with SEM-Bayesian are quite robust than the different prior inputs.

We could conclude that the statistics based on Bayesian SEM is not sensitive to these three different prior inputs. In other words, the Bayesian SEM technique applied here is quite robust than the different prior inputs. In the adolescent girl model, the standard errors of Type II prior were lesser than Type I and Type III priors; however, in the adolescent boy model, the standard errors of Type I prior were lesser than Type II and Type III priors. Therefore, we used the outputs involving Type II prior for the adolescent girl model and Type I prior for the adolescent boy model.

**Figure 3:**
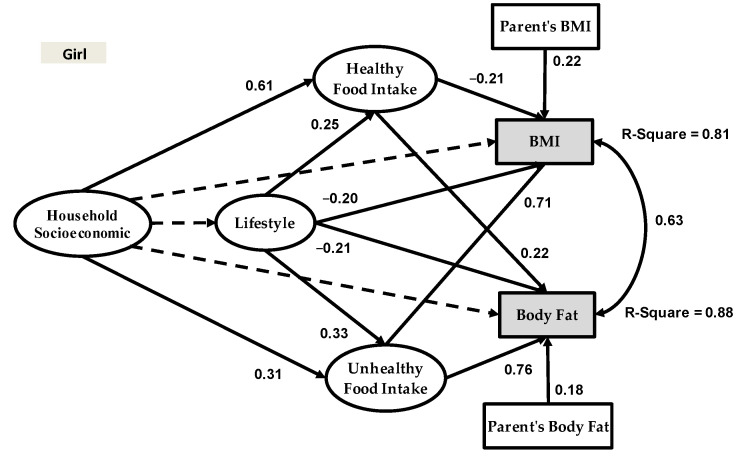
Adolescent girl obesity model.

**Figure 4:**
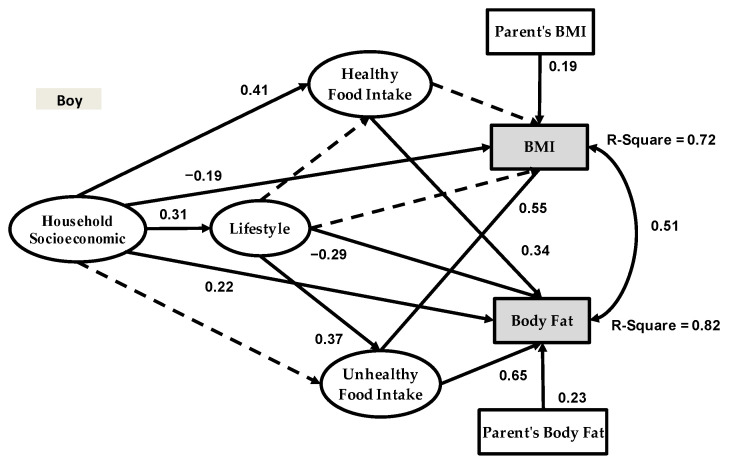
Adolescent boy obesity model.

Figure 3 and Figure 4 show the structural obesity models for adolescent girls and boys.

The model outputs for adolescent girls in Figure 3 reveal that household socioeconomic status had a significant and positive impact on healthy food intake (Beta = 0.61) and unhealthy food intake (Beta = 0.31), but no significant impact on lifestyle, BMI or body fat. Meanwhile, in the adolescent girl model, lifestyle had a significant impact on healthy food intake (Beta = 0.25), BMI (Beta = −0.20), body fat (Beta = −0.21) and unhealthy food intake (Beta = 0.33). However, the relationships among research variables in the adolescent boy model were different than those in the adolescent girl model. Based on Figure 4, household socioeconomic had a significant impact on healthy food intake (Beta = 0.41), BMI (Beta = −0.19), lifestyle (Beta = 0.31), and body fat (Beta = 0.22); however, this variable had no significant impact on unhealthy food intake. Meanwhile, in the adolescent boy model, lifestyle had a significant impact on body fat (Beta = −0.29) and unhealthy food intake (Beta = 0.37); however, this variable had no significant impact on healthy food intake and BMI.

### 3.3. Moderation Analysis

**Figure 5:**
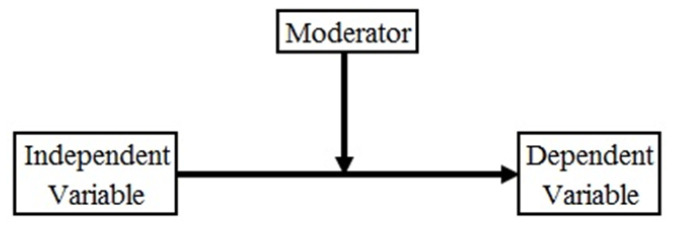
Moderating variable (Reprinted from Ref. [51]).

A moderator moderates the relationship between two variables (independent and dependent; cause and effect). Figure 5 graphically shows the impact of a moderator in the relationship between the dependent and independent variables. Furthermore, a moderator arises when it can influence the relationship between the two variables.

A moderator could either be a normal variable grouped in a study, such as age, time and gender, or a manipulated indicator in an experimental context or types of systems. In our study, we hypothesized that gender could be a moderator in any relationship between two variables, as presented in Figure 1. Correspondingly, we applied multi-group analysis to test the moderating effect of gender. Table 6 shows the multi-group outputs.

Out of fifteen relationships, eight of them had significant differences. Taken together, these data suggest that obesity modeling differed significantly between adolescent boys and girls. For instance, based on Table 5, the regression coefficients of “Household Socioeconomic → Healthy Food Intake” for the adolescent boy and girl models were 0.41 and 0.61, respectively, and the Z-score was 2.54, which is bigger than 1.96. Therefore, gender can serve as a moderator in the relationship between Household Socioeconomic and Healthy Food Intake. Furthermore, the differences in the relationship between Household Socioeconomic and Healthy Food Intake between the adolescent boy and girl models were significant. Statistically, the regression coefficients of Household Socioeconomic → Healthy Food Intake in the adolescent girl model had a significant difference from the adolescent boy model. 

## 4. Discussion

The present obesity prevalence among Iranian adolescents requires evidence-based intervention programs to combat obesity [52]. Obese adolescents are at the greatest risk of becoming obese adults, and numerous studies have documented the economic burden of obesity, including its comorbidities to society. Furthermore, recent data have shown a statistically significant number of obese adolescents who are now diagnosed with obesity-related diseases such as high blood pressure, type 2 diabetes and cardiovascular diseases, which had only be seen before in adults.

The present study sought to analyze causal relationships among several factors, namely household socioeconomic status, eating behavior, lifestyle, BMI and body fat using SEM-Bayesian. The introduced framework for adolescent obesity modeling includes two dependent variables, namely BMI and body fat (Figure 1). Meanwhile, household socioeconomic status was selected as the main independent variable in this framework. Between household socioeconomic status and dependent variables, three mediating variables were defined in line with previous research on adolescent obesity modeling [27]. The proposed research framework was therefore designed according to the development of prior concepts and patterns of adolescent obesity modeling that was built upon household socioeconomic status and eating behavior.

To demonstrate the validity and accuracy of the proposed research framework, in light of suitable research variables and their relationships, sampling was conducted across adolescents in Tehran, Iran, and our sample size includes 881 respondents. The research framework is presented in Figure 1. We extracted two categories of data based on gender from the dataset, and subsequently developed two separate models for adolescent boys and girls. The results for both gender groups are reported in Figure 3 and Figure 4. As shown in Figure 1, the research models examined two dependent variables and we had two R-squared values for each model. Based on SEM-Bayesian outputs, the R-squared values for the adolescent girl obesity model in terms of BMI and body fat were 0.81 and 0.88, respectively. It is important to note that 88% of the body fat variation was associated with household socioeconomic status, healthy food intake, unhealthy food intake and physical activity. The results also revealed that 81% of the BMI variation was associated with the same variables. However, the R-squared values for the adolescent boy obesity model were 0.82 for body fat and 0.72 for BMI, which are lower than the adolescent girl obesity model. 

Results of the present study are consistent with previous literature that has examined the relationship between household socioeconomic status and BMI and body fat [53,54,55]. However, diverging from the previous studies that omitted gender in their models, the results of the present study suggested that gender was a significant moderator in the adolescent obesity modeling equation. The present study further provides evidence that the relationship between household socioeconomic status and BMI and body fat was statistically significant for the adolescent boy obesity model; however, the same relationship did not hold true for the adolescent girl obesity model. In the adolescent girl obesity model, household socioeconomic status had an indirect effect on BMI and body fat through healthy food intake and unhealthy food intake, respectively. The data also suggested that parental education, household income and pocket money for adolescent boys are the significant variables in the household socioeconomic status latent variable (Table 6), impacting their BMI and body fat. Overall, these findings supported our hypothesis that gender might be a significant moderator in adolescent obesity modeling.

Lifestyle is the second independent variable in the present study, which includes screen time (TV, video games, laptop/PC and mobile phones), physical activity and sleep duration. Numerous studies have examined the relationships between obesity and adolescent lifestyle, including physical activity [56], sleep duration [57] and screen time. An early study by Simon and Kellou [58] found that regular physical activity was immensely useful to moderate the risk of obesity and prolong a healthy BMI in adolescents. In fact, leisure time through physical activity among adolescents has been recognized as a healthy lifestyle habit and promoted in developed countries [59]. Besides, recent studies by Misra and Grason [60] and Sainju and Manandhar [61] presented further evidence that physical activity increased during childhood and decreased during adolescence; however, this decline was more conspicuous in girls than in boys. Based on our results, the same held true for Iranian adolescent boys, where there was a significantly low level of physical activity among overweight and obese boys. Additionally, our results indicated that extended screen time was associated with increased BMI and body fat in adolescents. There is also evidence that extended screen time is indeed associated with unhealthy food intake (ranging from junk foods and soft drinks to fast food), and this in turn becomes a significant variable or factor contributing to obesity prevalence among adolescents. Lastly, another significant variable in our framework for adolescent obesity modeling includes sleep duration. In previous literature on adolescent obesity modeling, these three variables were examined as the main research variables (independent and mediator) by Turel and Romashkin [62] and as control variables by Khajeheian and Colabi [63] and Huang and Radzi [27]. Diverging from previous research, however, our study examined the combination of these three variables as the lifestyle latent variables in our new framework for adolescent obesity modeling.

Additionally, the most significant variable in this new framework for adolescent obesity modeling is body fat. This variable was examined as the second dependent variable; therefore, this new research framework has two dependent variables (i.e., BMI and body fat). The SEM-Bayesian results revealed that the R-squared of body fat in the adolescent girl obesity model was 0.81 and higher than the R-squared of BMI. On the contrary, in the adolescent boy obesity model, the same R-squared of body fat (0.82) was resultantly higher than the R-squared of BMI (0.72). Taken together, these results supported our hypothesis that the incorporation of body fat into our research framework would provide more insights into adolescent obesity modeling.

## 5. Conclusions

The present study sought to introduce a new framework for adolescent obesity modeling, taking into account complex multifactorial determinants such as household socioeconomic status, eating behavior and lifestyle that affect BMI and body fat. The findings of this study have important research implications, as follows:

1. This study is an improvement of previous research on adolescent obesity modeling because we introduced a new framework (Figure 1) that includes four latent variables and five measurement variables. Another novel contribution of the present study is the inclusion of body fat as the second dependent variable in our research framework—something that has never been done before. To our knowledge, the present study was the first to examine this relationship in adolescent obesity modeling.
2. The Bayesian estimator proposed the analysis of convenient structural equations for adolescent obesity modeling. In formulating SEM-ML and SEM-PLS in developing the Bayesian estimator, emphasis was placed on raw individual random observations rather than on the sample covariance and partial least square matrices.
3. The findings of the present study highlight the different structures of adolescent obesity modeling based on gender. As presented in Figure 3 and Figure 4, there were significantly different results from the SEM-Bayesian analysis between adolescent boys and girls.

However, the findings of the present study must be interpreted within the context of research limitations and important directions for future research:

1. Some other variables were not included in our research framework such as the economic, political and cultural determinants. They might, however, have a direct or indirect impact on household socioeconomic status and eating behavior. Furthermore, since our data were collected from Tehran, Iran, this makes the economic background a controlling factor in our research framework for adolescent obesity modeling. As such, our data may be representative of the urban Tehran population. Therefore, it is of particular importance that the proposed framework is employed in future research so that these findings can be replicated and extended in other areas and provinces across Iran and other countries.
2. Based on previous research, smoking [64,65], alcohol consumption [66,67,68] and genetics [69] were identified as significant indicators of obesity prevalence among adolescents, and hence should be included in the research framework. In the present study, however, we faced certain limitations to obtain data concerning these factors. For instance, more than 70% of participants did not respond to smoking habits and alcohol consumption. Therefore, we were not able to involve these two factors in our research model. Furthermore, genetic factors require DNA analysis based on a blood test that may incur high costs; however, we had a limited budget to involve a clinical test. Therefore, we suggest that future researchers include them in future studies in order to give more credence to our research framework and bolster the evidence of household socioeconomic status, lifestyle and eating behavior in adolescent obesity modeling.
3. The present study was limited to cross-sectional data structure and unable to determine any temporal associations between the variables of interest. Future research should thus incorporate longitudinal data that would permit more accuracy and confidence in data analysis with more definitive conclusions regarding adolescent obesity modeling.

## Figures and Tables

**Table 1:** Descriptive statistics of parents’ demographics.

Variable	Mother (Number)	Mother (Percentage)	Father (Number)	Father (Percentage)
**Age of Parents (years)**				
<31	163	19.6%	92	11.1%
31–40	271	32.6%	272	32.7%
41–50	240	28.9%	301	36.2%
51–60	142	17.1%	125	15.0%
>60	65	7.8%	91	11.0%
**Income of Parents (Million Toman)**				
<2	18	2.2%	11	1.3%
2–4	89	10.7%	47	5.7%
4–6	352	42.4%	381	45.8%
6–8	214	25.8%	198	23.8%
>8	208	25.0%	244	29.4%
**Education Level of Parents**				
Less than high school	32	3.9%	63	7.6%
High School	95	11.4%	98	11.8%
Diploma	366	44.0%	456	54.9%
Bachelor	301	36.2%	196	23.6%
Master or PhD	87	10.5%	68	8.2%
**Job Experience of Parents (years)**				
<5	29	3.5%	10	1.2%
5–10	267	32.1%	269	32.4%
11–15	324	39.0%	381	45.8%
16–20	209	25.2%	112	13.5%
>20	52	6.3%	109	13.1%

**Table 2:** Descriptive statistics of adolescent lifestyle.

Characteristics	Percentage		Characteristics	Percentage	
Physical Activity (per week)	Boy	Girl	Average sleep duration (hours per day)	Boy	Girl
None	28.1%	26.4%	Less than 7 h	7.8%	8.1%
1–2 times	38.3%	33.8%	7–8 h	47.9%	49.9%
3–4 times	22.0%	29.3%	8–9 h	35.2%	34.2%
More than 4 times	11.6%	10.4%	More than 9 h	9.1%	7.9%
Screen Time (hour per day)	Boy	Girl	Pocket Money (Toman per week)	Boy	Girl
Less than one hour	1.9%	2.0%	<100 k	13.0%	2.2%
1–2 h	26.7%	11.3%	101–150 k	34.3%	40.2%
3–4 h	27.4%	44.0%	151–200 k	29.0%	32.2%
More than 4 h	44.1%	42.7%	>200k	23.7%	25.3%

**Table 3:** Reliability and validity outputs.

Construct	Factor Loading	AVE	Cronbach’s Alpha
**Household Socioeconomic**			
Age of Father	0.48	0.54	0.72
Age of Mother	0.32		
Education of Father	0.79		
Education of Mother	0.71		
Income of Mother	0.46		
Income of Father	0.79		
**Lifestyle**			
Sleeping	0.81	0.55	0.77
Physical activity	0.83		
Screen time	0.79		
Pocket Money	0.87		
**Healthy Food Intake**			
Fruits	0.73	0.61	0.73
Vegetables	0.71		
Whole grains	0.76		
**Unhealthy Food Intake**			
Snacks	0.88	0.65	0.77
Fast Food	0.87		
Soft Drink	0.95		
Sweets	0.93		

**Table 4:** Comparative among of SEM-ML, SEM-PLS, and SEM-Bayesian outputs.

Indices	SEM	SEM	SEM	Function
	(ML)	(PLS)	(Bayesian)	
R^2^	0.73	0.68	0.78	$R^{2}=\frac{{\left[\sum_{i=1}^{n}\left(y_{i}^{\prime }-{\bar{y}}_{i}^{\prime }\right).\;\;\left(y_{i}-{\bar{y}}_{i}\right)\right]}^{2}}{\sum_{i=1}^{n}\left(y_{i}^{\prime }-{\bar{y}}_{i}^{\prime }\right).\;\;\sum_{i=1}^{n}\left(y_{i}-{\bar{y}}_{i}\right)}$
RMSE	1.304	2.362	1.229	$RMSE=\sqrt[{2}]{\frac{\sum_{i=1}^{n}{\left(y_{i}^{\prime }-y_{i}\right)}^{2}}{n}}$
MAPE	0.872	0.822	0.721	$MAPE=\frac{1}{n}\;\overset{n}{\underset{i=1}{\sum }}\left|\frac{y_{i}^{\prime }-y_{i}}{y_{i}}\right|$
MSE	0.087	0.101	0.081	$MSE=\frac{\sum_{i=1}^{n}{\left|y_{i}^{\prime }-y_{i}\right|}^{}}{n}$

**Table 5:** Evaluated Bayesian estimators based on three different priors.

Hyperarameter	Type I Prior		Type II Prior		Type III Prior	
	Estimate	S.E	Estimate	S.E	Estimate	S.E
**Girls’ Model**						
Household Socioeconomic → BMI (β_1_)	0.12	0.168	0.10	0.163	0.16	0.174
Household Socioeconomic → Body Fat (β_2_)	0.09	0.233	0.09	0.228	0.61	0.335
Lifestyle → BMI (β_3_)	−0.18	0.154	−0.20	0.134	0.57	0.189
Lifestyle → Body Fat (β_4_)	−0.24	0.166	−0.21	0.167	0.51	0.195
Healthy Food Intake → BMI (β_5_)	−0.23	0.185	−0.21	0.179	0.14	0.188
Healthy Food Intake → Body Fat (β_6_)	0.19	0.173	0.22	0.166	0.61	0.189
Unhealthy Food Intake → BMI (β_7_)	0.69	0.281	0.71	0.276	0.57	0.283
Unhealthy Food Intake → Body Fat (β_8_)	0.76	0.135	0.76	0.139	0.51	0.156
**Boys’ model**						
Household Socioeconomic → BMI (β_1_)	−0.19	0.231	−0.20	0.239	−0.19	0.288
Household Socioeconomic → Body Fat (β_2_)	0.22	0.115	0.25	0.214	0.21	0.156
Lifestyle → BMI (β_3_)	−0.09	0.211	−0.07	0.227	−0.09	0.235
Lifestyle → Body Fat (β_4_)	−0.29	0.201	−0.24	0.222	−0.26	0.229
Healthy Food Intake → BMI (β_5_)	0.09	0.105	0.08	0.118	0.11	0.108
Healthy Food Intake → Body Fat (β_6_)	0.34	0.149	0.36	0.151	0.31	0.169
Unhealthy Food Intake → BMI (β_7_)	0.53	0.207	0.51	0.208	0.57	0.216
Unhealthy Food Intake → Body Fat (β_8_)	0.65	0.163	0.66	0.166	0.62	0.175

**Table 6:** Multi-group analysis between the adolescent boy and girl models.

Path	Estimated Coef for Boy	Estimated Coef for Girl	Z-Score	*p*-Value
Household Socioeconomic → Healthy Food Intake	0.41	0.61	2.54	0.011
Household Socioeconomic → Unhealthy Food Intake	0.08	0.31	3.11	0.002
Household Socioeconomic → Lifestyle	0.31	0.05	3.36	<0.001
Household Socioeconomic → BMI	−0.19	0.10	4.06	<0.001
Household Socioeconomic → Body Fat	0.22	0.09	1.88	0.060
Lifestyle → Healthy Food Intake	0.06	0.25	2.43	0.015
Lifestyle → Unhealthy Food Intake	0.37	0.33	0.45	0.652
Lifestyle → BMI	−0.09	−0.20	1.57	0.116
Lifestyle → Body Fat	−0.29	−0.21	1.04	0.298
Healthy Food Intake → BMI	0.09	−0.21	4.23	<0.001
Healthy Food Intake → Body Fat	0.34	0.22	1.65	0.098
Unhealthy Food Intake → BMI	0.53	0.71	2.22	0.026
Unhealthy Food Intake → Body Fat	0.65	0.76	1.57	0.116
Parent’s BMI → BMI	0.19	0.22	0.32	0.748
Parent’s Body Fat → Body Fat	0.23	0.18	0.76	0.447

## Data Availability

The data presented in this study are available on request from the **c**orresponding author. The data are not publicly available due to the decision of the ethic committee of Statistical Center of Iran.

## Supplementary Materials

The following is available online at https://www.mdpi.com/article/10.3390/healthcare9080925/s1, Table S1: Terms and conditions of sample size in SEM.
