# Supplementary material for: Adolescent Obesity Modeling: A Framework of Socio-Economic Analysis on Public Health
Source: Healthcare (Basel). 2021 Jul 22;9(8):925. doi: 10.3390/healthcare9080925 (PMC8392515; doi:10.3390/healthcare9080925)
Supplement: Supplementary file 1 [file healthcare-09-00925-s001.zip › healthcare-1243365-supplementary.pdf]

Supplementary Table S1

Terms and conditions of sample size in SEM

| Model Characteristics (Number of latent constructs and items) | Sample Size Required |
|---|---|
| 1. Model includes five or less latent variables and each latent variable admits at least three indicators. | At least 100 samples |
| 2. Model includes seven or less latent variables and each latent variable admits at least three indicators. | At least 150 samples |
| 3. Model includes seven or less latent variables and some latent variables admit less than three indicators. | At least 300 samples |
| 4. Model includes more than seven latent variables and some latent variables admit less than three indicators. | At least 500 samples |
